# Emergency Department presentations due to new psychoactive substances (NPS) and other illicit drugs: a clinical and toxicological study on recreational drug toxicity in Italy

**DOI:** 10.1007/s00414-026-03795-0

**Published:** 2026-04-07

**Authors:** Arianna Giorgetti, Annamaria Venturi, Rossella Barone, Francesca Rossi, Filippo Pirani, Jennifer Paola Pascali, Fabrizio Giostra, Guido Pelletti

**Affiliations:** 1 Department of Medical and Surgical Sciences, Unit of Legal Medicine, University of Bologna, Bologna, Italy; 2 Emergency Department, IRCCS Azienda Ospedaliero-Universitaria Di Bologna, Bologna, 40138 Italy; 3 Medicina Legale E Risk Management, Azienda USL Di Bologna, Bologna, Italy

**Keywords:** Forensic toxicology, Recreational drugs, Novel psychoactive substances, Ketamine, Cathinones

## Abstract

**Abstract:**

Although the use of new psychoactive substances (NPS) has increased worldwide, current drug monitoring systems in emergency departments (EDs) across several European countries including Italy, remain mainly focused on classical drugs of abuse. This study aimed to assess the prevalence of NPS, ketamine and other illicit drugs in patients presenting to ED in Bologna, Italy, collecting associated clinical and toxicological data.

**Methods:**

This observational study included patients presenting to the ED of the University-Hospital of Bologna (IRCCS Azienda Ospedaliero-Universitaria di Bologna, Italy), between March and November 2024, with suspected acute recreational drug toxicity. Patient demographics and clinical features were recorded, and blood samples were analyzed for NPS and other drugs, using a previously validated and updated liquid chromatography-tandem mass spectrometry (LC–MS/MS) method.

**Results:**

Of the 110 patients enrolled, 67 (60.9%) tested positive for at least one drug. Cocaine was the most frequently detected substance (*n* = 50; 74.6% of positive cases). Twenty patients tested positive for ketamine or norketamine (25.4% of positive cases), with mean values of 55 ng/ml and 101 ng/ml, respectively. NPS were detected in 3 patients (4.5% of positive cases) and consisted of methylone in 2 cases (below the limit of quantification), alpha-PHP (below the limit of quantification) and 3,4-MD-alpha-PHP in one case (16 ng/ml).

**Discussion:**

Our study reveals a high prevalence of ketamine use, and a predominance of stimulants, particularly synthetic cathinones, among NPS. The association of NPS with psychomotor agitation underscores the clinical importance of considering these substances in cases of severe agitation. These findings emphasize the evolving landscape of recreational drug use and the critical role of comprehensive toxicological screening in emergency settings.

## Introduction

New Psychoactive Substances (NPS) represent a wide and diverse group of substances designed to mimic the effects of traditional drugs while evading legal regulations. The European Union Drug Agency (EUDA) has identified over 1000 NPS, categorized into subgroups, namely synthetic and semi-synthetic cannabinoids, synthetic cathinones, synthetic opioids, designer benzodiazepines, and tryptamines. NPS often exhibit higher potency and unpredictability compared to traditional drugs of abuse, posing significant risks of severe acute intoxications and fatalities [1].

The analytical detection of NPS is challenging due to their rapid emergence and structural diversity, which complicates the development of effective screening tests [2]. Traditional immunoassays commonly used in emergency departments (EDs) are ineffective for NPS detection, leading to potential underdiagnosis [3]. Advanced techniques like liquid chromatography-tandem mass spectrometry (LC–MS/MS) or high-resolution mass spectrometry (HRMS) are required for accurate identification but are not typically available in clinical settings [4, 5].

Epidemiological parameters, such as the prevalence of use and abuse, are crucial for estimating burden metrics that guide healthcare priorities and service planning. Epidemiological data on NPS often come from surveys conducted via questionnaires, internet forums, or calls to poison information centers [6]. Recent studies have explored the prevalence of NPS among specific subpopulations worldwide, revealing that these substances are more commonly used by young individuals or those frequenting nightclubs [7] and music festivals [8], mentally ill patients [9], homeless individuals [10], or people in prison [11]. Recently, the prevalence among individuals diagnosed with substance use disorder in Italy has also been assessed, revealing emerging patterns of polydrug consumption [12]*.* Moreover, NPS might be used as adulterants of drugs of abuse, leading traditional drugs consumers to unexpected exposures [1, 6, 13].

To date, few studies have investigated the prevalence of NPS in patients presenting to EDs with analytical confirmation [14–18]. Although self-reporting can be a useful tool for estimating prevalence, only analytical confirmation, which is not always possible in an ED, provides certainty about the presence of substances and allows to study patterns of consumption.

This study aims to investigate the prevalence of NPS among patients presenting with suspected acute recreational drug toxicity at the ED of the University-Hospital of Bologna (IRCCS Azienda Ospedaliero-Universitaria di Bologna, Italy). The objectives include the qualitative and quantitative determination of NPS and other illicit drugs, the identification of consumption patterns with respect to gender, age and substance subgroups together with the collection of clinical and toxicological data.

## Materials and methods

### Study design

All presentations at the ED occurred between 1 March and 30 November 2024, that were suspected of acute intoxication, were included. Patients were enrolled when the attending emergency physician suspected acute recreational drug toxicity, either on the basis of self-reported recreational drug use or clinical presentations consistent with acute toxicity from recreational drug abuse, such as unexplained changes in consciousness, agitation, acute behavioral changes, hallucinations, suicide attempts or non-suicidal self-harm. As all patients routinely undergo blood sampling at the ED for clinical purposes, after acquiring written consent, an additional blood sample was collected specifically for this study. The study-specific samples were stored at −20 °C until further toxicological evaluation.

The study, observational in nature, was approved by the Ethics Committee Area Vasta Emilia Centro (AVEC) approval n° 582/2023/Oss/AOUBo, date of approval 13/09/2023.

### Clinical characteristics and extracted data

For each presentation, the following data were extracted: demographic data (date and time of presentation, age, gender); assigned color code at the triage; chief complaints, including self-report of drug of abuse; vital signs at triage including Glasgow coma scale (GCS), blood pressure, respiratory rate, saturation, temperature; clinical features recorded by the physician and time of clinical evaluation; past history, including psychiatric disease or past drug abuse; drugs administered by the Emergency Medical Service or at hospital. The delay between ED presentation and blood sampling was assessed.

Drugs detected in the blood samples that were confirmed to be administered by the Emergency Medical Service or by emergency physicians at the hospital were excluded from statistical analyses.

### Analytical investigation

Blood samples were analyzed by liquid chromatography tandem mass spectrometry (LC–MS/MS) at the Laboratory of Forensic Toxicology of the University of Bologna. Analyses were performed using a Waters Acquity (Ultra High‐Performance Liquid Chromatography) UHPLC® (Milford, MA) coupled to a triple quadrupole mass detector Waters Xevo TQD, using electrospray ionization (ESI) in positive mode and multiple reaction monitoring mode (MRM).

Chromatographic separation was achieved using an Acquity UPLC® HSS C18 column (1.8 μm, 2.1 × 150 mm from Waters, Milan, Italy).

Identification and quantification of 182 NPS were performed on all samples, through a previously developed and validated method, monitoring synthetic cannabinoids (SCs), synthetic opioids (SOs), synthetic cathinones (SCAs), stimulants and other drugs [19], updated to cover novel molecules.

The method was also updated to include qualitative data on traditional drugs of abuse and metabolites: cocaine, benzoylecgonine, ecgonine methyl ester (EME), cocaethylene, amphetamine, methamphetamine, 3,4-methylenedioxyamphetamine (MDA), 3,4-methylenedioxymethamphetamine (MDMA), morphine, 6-monoacetylmorphine, codeine, methadone, 2-ethylidene-1,5-dimethyl-3,3-diphenylpyrrolidine (EDDP), delta-9-tetrahydrocannabinol (delta-9-THC). Internal standards solution contained nordiazepam‐D5, ketamine‐D4 and fentanyl-D5.

The updated method was applied as a broad screening tool for a wide panel of NPS and traditional drugs of abuse. Upon identification of samples testing positive for previously non-validated compounds (e.g. norketamine, alpha-PHP and 3,4-MD-alpha-PHP), targeted validation was performed for the detected analytes. Method validation took place as reported for the other 182 NPS [19] and results are reported in Table 1.

**Table 1:** Validation results for 3,4-MD-alpha-PHP, alpha-PHP ketamine, norketamine, and methylone

Compound	R^2^ with range 0.25–10 ng/ml	Equation	LLOQ	LOD	QC low (0.5 ng/ml)	QC high (10 ng/ml)
3,4 MD-alfa-PHP	0.999	1.3882x-0.0711	0.25 ng/ml	0.08—0.1 ng/ml	bias: **5.45%** IntraCV: **1.69%** InterCV: **2.01%**	bias:** 8.71%** IntraCV:** 0.18%** InterCV:** 0.87%**
alfa-PHP	0.9993	4.304x + 0.3889	0.25 ng/ml	0.08—0.1 ng/ml	bias: **14.56%** IntraCV: **1.48%** InterCV: **3.30%**	bias: **11.88%** IntraCV: **1.10%** InterCV: **1.26%**
ketamine	0.9978	2.1197x + 0.1044	0.25 ng/ml	0.08—0.1 ng/ml	bias: **12.17%** IntraCV: **1.91%** InterCV: **2.08%**	bias: **5.76%** IntraCV: **0.83%** InterCV: **1.25%**
norketamine	0.9971	1.1036x + 0.0137	0.25 ng/ml	0.08—0.1 ng/ml	bias: **8.73%** IntraCV: **2.50%** InterCV: **3.30%**	bias: **5.10%** IntraCV: **1.14%** InterCV: **1.27%**
methylone	0.9951	1.1929x + 0.1016	0.25 ng/ml	0.08—0.1 ng/ml	bias: **10.93%** IntraCV: **3.10%** InterCV: **1.59%**	bias: **8.64%** IntraCV: **1.62%** InterCV:** 2.54%**

*IntraCV* Intra-day coefficient of variation, *InterCV* interday coefficient of variation, *LOD* limit of detection, *LLOQ* lower limit of quantification, *QC* quality control

For traditional drugs of abuse, since the method was intended as a screening, the re-validation only focused on sensitivity, with the following limit of detections (LODs): 0.5 ng/ml for cocaine, benzoylecgonine, EME, cocaethylene, amphetamine, methamphetamine, MDA, MDMA, morphine, 6-monoacetylmorphine, codeine, methadone and EDDP; 2.5 ng/ml for delta-9-THC.

When samples exceeded the upper quantification limit (ULOQ), dilution integrity testing (2x, 10x, 20x) showed acceptable results (± 15% bias and ≤ 15% coefficient of variation), so samples were diluted before analysis. In similar cases, concentrations were given as approximated to whole numbers, avoiding decimal values.

### Data analysis

Positivity to cocaine was reported when cocaine, together with at least one metabolite, was detected in blood. Positivity to amphetamines/methamphetamines was reported when at least one compound among amphetamine, methamphetamine, MDA, and MDMA was detected in blood. Positivity to methadone when assigned when methadone and EDDP were detected in blood. Positivity to other opioids (morphine/heroin/codeine) was reported when at least one among morphine, 6-monoacetylmorphine and codeine was identified.

For statistical analyses, the total cohort was classified into three groups: the NPS-positive group (all cases in which NPS were detected alone or in addition to traditional illicit drugs of abuse), the ketamine-positive group (all cases in which ketamine or norketamine were detected alone or in addition to traditional illicit drugs of abuse), and the other illicit drug group.

Patient demographics and clinical data were compared between the NPS-positive group and the other illicit drug group as well as between the ketamine-positive group and the other illicit drug group.

Descriptive statistics were provided for all data, with a focus on cases with drug detection at blood analysis (positive cases), including mean and standard deviation (SD) and/or median and interquartile range (IQR).

When comparing quantitative data, parametric or non-parametric t-test were performed, according to the results of the sktest, a normality test that considers skewness and kurtosis.

When comparing qualitative data, the chi-square test was performed.

Statistical analysis was performed with Stata (StataCorp v. 15.1, 4905 Lakeway Drive, College Station, Texas 77,845 USA). Figures were created with Prism (v. 10.2.1 GraphPad Software, LLC) and Flourish (Canva, UK Operations Ltd, UK 33, Hoxton Square, London N1 6NN).

## Results

During the 9-month period of the study, a total of 110 patients were enrolled. The patients were mostly men (68.2%) with a mean age of 31.7 years (SD = 12.4). Chief complaints included: agitation and behavioral changes (*n* = 47, 42.7%), altered mental status (*n* = 24, 21.8%), recreational drugs use with non-specific symptoms (*n* = 19, 17.3%), cardiothoracic complaints (*n* = 12, 10.9%) and other complaints (*n* = 8, 7.3%).

Thirty-eight patients reported using drugs: 18 patients reported using cocaine (16.4%), 7 ketamine (6.4%), 7 cannabis (6.4%), 4 stimulants or amphetamines (3.6%) and 2 reported using heroin (1.8%).

The assigned color code at triage was mostly orange (*n* = 55, 50%), followed by red codes, assigned in 25 cases (22.7%). Blue (*n* = 14, 12.7%), green (*n* = 13, 11.8%) or white (*n* = 3, 2.8%) codes were less frequently assigned.

### Demographics of cases with positive drug detection

**Fig. 1:**
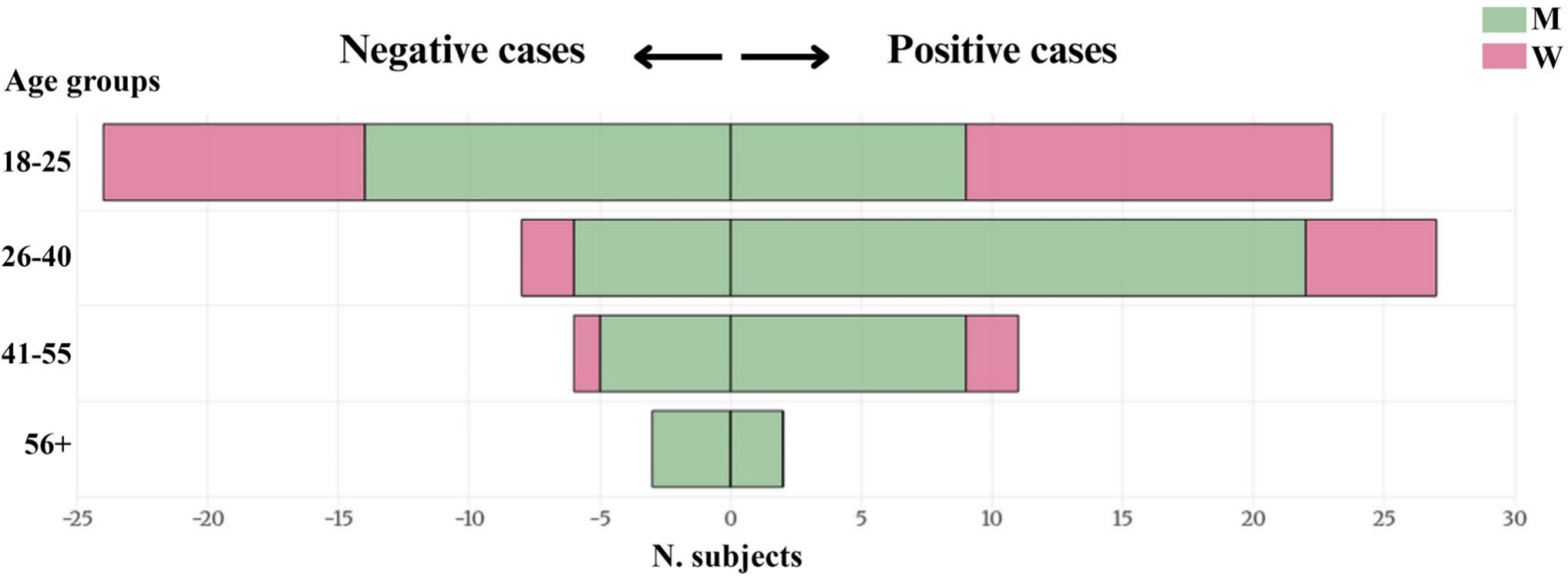
Demographic characteristics of cases with positive (on the right, positive values) and negative (on the left, negative values) drug detection. W: women, in pink, M: men, in green

At least one drug was detected by LC–MS/MS analysis in blood samples in 67 patients, representing 60.9% of the total cohort (positive cases). Among these, the majority were men (67.2%), and the mean age was 32.9 years (SD = 11.6). Women were younger than men, with a mean age 28.1 years (SD = 10.5) compared to 35.4 years (SD = 11.5) for men, and this difference was statistically significant (*p* = 0.015). Figure 1 provides a more detailed description of demographics by age groups.

### Substances detected and delay

Among the 67 positive cases, the most frequently detected traditional drug of abuse was cocaine (*n* = 50, 74.6%). Less frequently detected were methamphetamine/amphetamines (including MDA and MDMA) (*n* = 13, 19.4%), methadone (*n* = 12, 17.9%), other opioids (morphine/heroin/codeine) (*n* = 11, 16.4%) and delta-9-THC (*n* = 4, 6.0%).

Ketamine or norketamine was detected in 20 cases, including 3 cases of administration by the Emergency Medical Service, which were excluded from further analyses, and 17 cases of self-administration (25.4% of positive cases). Of the ketamine-positive cases, 12 additionally tested positive for other illicit drugs. Ketamine-positive presentations particularly tested positive for cocaine (*n* = 9, 52.9%), amphetamines/methamphetamines (*n* = 9, 52.9%), methadone (*n* = 2, 11.8%), other opioids (morphine/heroin/codeine) (*n* = 1, 5.9%) and delta-9-THC (*n* = 2, 11.8%).

Among the ketamine-positive group, ketamine and norketamine were below LLOQ in 7 out of 17 cases (41.2%), above LLOQ and below ULOQ in 3 (17.6%), and above ULOQ in 7 cases (41.2%). The latters were diluted to proceed to further quantification.

Mean approximate ketamine concentration was 55 ng/ml (SD = 52), median 34 ng/ml (IQR = 5–108), with minimum and maximum concentrations of 0.65 ng/ml and 125 ng/ml; mean approximate norketamine concentration was 101 ng/ml (SD = 93), median 74 ng/ml (IQR = 15–158), with minimum and maximum concentrations of 0.96 ng/ml and 250 ng/ml.

NPS were detected in 3 cases (4.5% of positive cases) and consisted exclusively of 3 synthetic cathinones: methylone (*n* = 2, 3.0%), alpha-PHP (*n* = 1, 1.5%), and 3,4-MD-alpha-PHP (*n* = 1, 1.5%). NPS were only detected in cases that were also positive for other illicit drugs, particularly cocaine (*n* = 2, 66.7%) and amphetamines/methamphetamines (*n* = 2, 66.7%), but never methadone, other opioids or delta-9-THC.

Methylone was below LLOQ in both cases. Alpha-PHP was also below LLOQ, while 3,4-MD-alpha-PHP was quantified after dilution at 16 ng/ml.

In positive cases, the mean delay between presentation and blood sampling was 1.1 h (SD = 1.6), the median was 0.4 h (IQR = 0.15–1.2), and the maximum reported delay was 7 h. In negative cases, the mean delay was 1.5 h (SD = 3.7), with a median of 0.5 h (IQR = 0.2–0.9); no significant difference in delay was found between negative and positive cases.

Similar results were observed for ketamine-positive cases, with a mean delay of 0.9 h (SD = 1.7), and for NPS-positive cases, with a mean delay of 0.3 h (SD = 0.2), so that differences were not statistically significant.

### Clinical features of cases with positive drug detection

In positive cases, chief complaints mostly included agitation and behavioral changes (*n* = 26, 39.4%), followed by altered mental status (*n* = 17, 25.8%), and recreational drugs use (*n* = 12, 18.2%). Less frequently, cardiothoracic complaints (*n* = 9, 13.6%) and other symptoms (*n* = 2, 3.0%) were reported.

Among cases tested positive for any drug at LC–MS/MS analysis of blood samples, most manifested psychomotor agitation (41.8%) or depressed consciousness (38.8%), followed by cardiac complaints (22.4%), disorientation/confusion (16.4%) and aggression/violence (14.9%).

The clinical features are summarized together with vital parameters at triage in Table 2.

**Table 2:** Clinical features of cases with positive drug detection in blood samples. bpm: beats per minute

	Number of cases (n)	Percentages
**Vital parameters at triage**		
Glasgow coma scale < 14	13	19.4%
Systolic blood pressure		
Low (< 90 mmHg)	2	3.0%
High (> 14 mmHg)	12	17.9%
Heart rate		
Bradycardia (< 60 bpm)	1	1.5%
Tachycardia (> 100 bpm)	13	19.4%
Respiration rate		
Tachypnea (> 20/min)	2	3.0%
Saturation		
Desaturation (< 94%)	6	9.0%
Temperature		
Fever	1	1.5%
**Pain/general symptoms**		
Cardiac complaints including dyspnea	15	22.4%
Abdominal pain	3	4.5%
General complaints (astenia/pain)	4	6.0%
**Neuropsychiatric symptoms**		
Psychomotor agitation	28	41.8%
Depressed consciousness	26	38.8%
Disorientation/confusion	11	16.4%
Anxiety	9	13.4%
Mood disorder	7	10.4%
Hallucinations	6	9.0%
Acute psychosis/delirium	5	7.5%
Bizarre behavior	3	4.5%
Hypoesthesia	1	1.5%
Amnesia	1	1.5%
Confabulation/involuntary movements	1	1.5%
Syncope	1	1.5%
**Other symptoms**		
Nausea/vomiting	5	7.5%
Sweating	3	4.5%
Insomnia	2	3.0%
Tremors	2	3.0%
Morsus	1	1.5%
**Injury-related events**		
Aggression and violence	10	14.9%
Self-injury	5	7.5%
Skin injuries	4	6.0%
Trauma	2	3.0%
Suspected victim of abuse	1	1.5%

### Comparison between ketamine group, NPS group and other illicit drugs group

Ketamine-positive presentations involved slightly younger patients (mean age of 28.3 years, SD = 8.5) compared to the other illicit drugs group (mean age 34.4 years, SD = 12.2), but the difference was not statistically significant (*p* > 0.05). In contrast, a statistically significant association was found between ketamine positivity and gender, with ketamine-positive presentations involving a larger proportion of women than cases positive for other illicit drugs (*p* = 0.041).

Assigned color codes and chief complaints were not statistically associated with the ketamine-positive group (*p* > 0.05). Additionally, when considering the main clinical features (psychomotor agitation, depressed consciousness, anxiety, mood disorder, cardiac complaints, aggression and violence), no significant difference in frequency was detected between ketamine-positive cases and other illicit drugs-positive cases (*p* > 0.05). Presentations to the ED were almost equally motivated by agitation and behavioural changes (29.5%), altered mental status, recreational drugs use with non-specific symptoms and cardiothoracic complaints (23.5% each). Among ketamine-positive presentations, 4 cases manifested aggression and violence (23.5%), and 2 cases involved self-injury (11.8%).

When comparing the ketamine-positive group with the other drugs-positive group, a larger proportion of ketamine-positive cases tested negative for cocaine (*p* = 0.017). In contrast, the ketamine-positive group was more frequently positive for amphetamines/methamphetamines compared to the other illicit drugs-group (*p* = 0.001). Associations with the other illicit drugs tested non-significant.

**Fig. 2:**
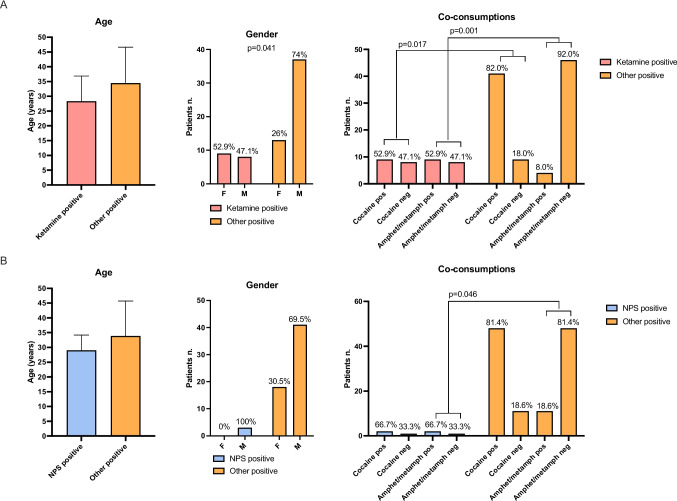
Age, gender and co-consumption patterns of ketamine-positive cases (**A**) and NPS-positive cases (**B**). Statistically significant associations are reported with their p-value

Age, gender, and co-consumption patterns of ketamine-positive presentations are shown in Fig. 2A.

NPS-positive presentations did not differ in age compared to other illicit drugs-related presentations and were all men, resulting in a non-significant association with gender (*p* > 0.05). NPS-positive cases were either classified as orange or red at the color-code triage assignment, although this association was also non-significant.

NPS-positive presentations showed a higher rate of positivity for amphetamines/methamphetamines compared to the other illicit drug group, with *p* = 0.046. Age, gender, and co-consumption patterns of NPS-positive presentations are shown in Fig. 2B.

Chief complaints did not differ between NPS-group and other illicit drugs-positive cases (*p* > 0.05). When considering clinical features, all NPS-positive patients presented psychomotor agitation (100%, vs 39.0% of other illicit drugs) and the association was statistically significant (*p* = 0.037), while other symptoms showed non-statistically significant associations.

When considering the main clinical features (psychomotor agitation, depressed consciousness, anxiety, mood disorder, cardiac complaints, aggression and violence), no difference in frequency was detected between the ketamine-positive group and the other illicit drugs positive group (*p* > 0.05).

Clinical and toxicological data of the NPS-positive cases are shown in detail in Table 3.

**Table 3:** Age, gender (M: men, W: woman), assigned color code, triage parameters, chief complaints, symptoms, clinical history, administered drugs and results of toxicological analysis for the NPS-positive group

	Age (years)	M/W	Delay (h)	Assigned code – triage parameters	Chief complaint	Symptoms and history	Administered drug	NPS	Other drugs/metabolites detected
NPS-positive case #1	35	M	0.47	Orange – triage parameters not reported	Psychomotor agitation	Paranoid agitation with hallucinations, accelerated speech, aggressive behaviour Past consumption of hallucinogens and party drugs	Diazepam, haloperidol, clotiapine, delorazepam, promazine	3,4-MD-alpha-PHP (16 ng/ml), alpha-PHP < LLOQ	Cocaine, BEG, EME
NPS-positive case #2	26	M	0.13	Red – triage parameters not reported	Depressed consciousness	Closed eyes, but arousable to verbal command, then psychomotor agitation	Delorazepam	Methylone < LLOQ	Cocaine, BEG, EME, cocaethylene, MDA, MDMA
NPS-positive case #3	26	M	0.38	Red – Blood pressure: 200/80 mmHg, heart rate 140 bpm, temp 37.5 °C	Psychomotor agitation	Agitation with aggressive behaviour, sweating, tachycardia, skin injuries at lower legs	Midazolam	Methylone < LLOQ	MDA, MDMA, norketamine

*LLOQ* lower limit of quantification

## Discussion

Although the focus of the EUDA and international organisms has long been mainly on NPS [1], only a few studies to date have attempted to define their prevalence among patients attending the ED, in order to understand patterns of recreational drug use alongside the characteristics of users. The present study, the first conducted in Italy, reports the analytical identification of drugs of abuse and NPS in a population of suspected acute poisonings presenting to the ED.

The demographics of positive cases (positivity to at least one drug) aligned with findings from similar studies, which often report a predominance of male patients and a mean age in the early 30 s [20, 21]. Our data on drugs of abuse showed a clear prevalence of cocaine over all other drugs (74.6%). This confirms findings from previous Italian studies, both in intoxicated drivers [22] and in acute fatal intoxications [23], highlighting the very high prevalence of cocaine users in our country [12]. The 19.4% prevalence of methamphetamine/amphetamines in our study population exceeds that of substances more commonly detected in our country, such as methadone and cannabis. This unexpected prominence is likely attributable to the acute systemic symptoms that prompted patients to seek emergency care, also considering the significant association of these substances with ketamine and NPS.

One of the main findings of the study is the very high percentage of ketamine-positive patients (17 cases in total, 25.4% of positive cases), involving a larger proportion of women than cases involving other illicit drugs and less frequently associated with cocaine. Ketamine is a well-known and relatively safe anesthetic drug, but is also a recreational drug classified as a NPS by the United Nations Office on Drugs and Crime (UNODC) [24].

From 2009 to 2019 in Italy, ketamine abusers represented 9.4 per 1,000 presentations to 11 EDs, most often involving males [25]. Although it is difficult to compare prevalence with previous literature due to different methodologies or settings, our results suggest a growing spread of the substance in recent years, as well as a closing of the gender gap – both novel findings in Italy.

A higher frequency of ketamine-positive patients compared to the past has recently been observed in Taiwan by Lin et al. [15] but our prevalence is still higher compared to the data from EDs of other countries [14, 17, 20].

The rise in ketamine use has been associated with the consumption by young adults in nightlife settings [8], a finding supported by our study through the statistically significant association with amphetamines/methamphetamines.

Nevertheless, a high presence of ketamine has also recently been observed in patients with substance use disorder [12], possibly due to ketamine being used as a cutting agent for MDMA, cocaine and heroin.

This trend highlights the complexity of managing acute drug toxicity in emergency settings and underscores the need for comprehensive toxicological screening. This high frequency of ketamine intoxication poses significant treatment challenges in emergencies, as ketamine—significantly associated with other synthetic drugs such as amphetamines/methamphetamines and NPS in this study–is often administered for sedation or intubation procedures [26]. Given the widespread recreational use of ketamine, it is important to consider any potential prior consumption when selecting the most appropriate treatment for each patient.

Regarding NPS, only 3 patients tested positive, all presenting with psychomotor agitation, for a total of 4 positive results, since case 1 was positive for both 3,4-MD-alpha-PHP and alpha-PHP.

3,4-MD-alpha-PHP is a synthetic cathinone known to cause severe toxidromes typically characterized by psychomotor agitation and aggressiveness [27]. According to seizures and data from the National Early Warning System on Drugs, it currently dominates the Italian black market for NPS [28]. Alpha-PHP has been linked to mono or, more frequently, polydrug intoxications [29, 30]; and, in our NPS-positive case #1, the combination of multiple stimulants could have contributed to the aggressive and violent behaviour observed.

Methylone, a first-generation synthetic cathinone frequently seized around 2013–2015 in Italy [31], can lead to severe toxicity including hyponatremia, seizures and MDMA-like effects [32]. In NPS-positive cases #2 and #3, methylone may have contributed to the reported symptoms, although low concentrations suggest it could also have been used as a contaminant or cutting agent of traditional drugs of abuse.

Published studies available for comparison, providing analytical confirmation of NPS in patients presenting to the ED are limited in number and often report small sample sizes. The percentage of NPS-positive cases in our study is much lower than in similar studies conducted in Australia [17] or Asia [16] with comparable design and populations, once again highlighting the impact of regional variations and drug availability in drug prevalence. A recent European study [33] observed a very high percentage of synthetic cannabinoids (41.4%) and a reduced percentage of cathinones (*n* = 4, 3.5%), possibly due to the exclusion criteria (clinical symptoms of intoxication other than NPS or other classical of illicit drugs). Similarly to our results, a low prevalence of NPS, with a predominance of synthetic cathinones, especially in association with stimulants, was reported in Switzerland and Spain [20, 34]. Based on these findings, in cases of psychomotor agitation, particularly among patients positive for amphetamines/methamphetamines, clinicians should raise suspicion of synthetic cathinones or ketamine use.

The limited time frame and monocentric nature of our study do not allow us to generalize the prevalence of NPS to the entire Italian population. However, the absence of synthetic benzodiazepines, synthetic opioids, or synthetic cannabinoids – substances more commonly reported in international case studies–is unexpected. While synthetic drug use in Italy is indeed lower than in other European countries [35], this finding may also reflect limitations in the study design. In particular, the time elapsed between substance intake and blood sampling in many cases could have led to undetected compounds with short half-lives. This is an important aspect of the study that requires improvement, with efforts aiming to reduce the time from patient presentation to blood sampling in the ED. Moreover, only a limited set of substances was tested, although wide and covering most NPS subgroups, but updated analytical methods would be required for further research. Lastly, in cases of samples above ULOQ, a dilution of samples was performed, and results were approximated to whole numbers to avoid decimal values. Though an exact quantification was beyond the scope of our analysis, wider ranges will be considered, especially for ketamine and norketamine, in future work.

Nevertheless, the data collected in this first Italian experience are not only meaningful for providing insights in the clinical and forensic setting, but also useful for guiding future research.

## Conclusions

The results of our study indicate a high prevalence of ketamine, likely reflecting regional variations in drug availability and preferences, which warrants careful consideration in the emergency department. Along with the exclusive detection of synthetic cathinones among NPS, these findings provide relevant epidemiological data for the forensic field, suggesting that stimulants and party-drugs are more prevalent in our Italian sample than synthetic cannabinoids or opioids.

Notably, all NPS-positive cases in our cohort presented with psychomotor agitation, a finding that was statistically significant compared with other illicit drug cases. This observation aligns with previous reports of NPS-associated agitation and emphasizes the importance of considering NPS in both clinical and forensic evaluations of cases of severe agitation.

## Data Availability

Not applicable.
